# SOX10, a novel HMG-box-containing tumor suppressor, inhibits growth and metastasis of digestive cancers by suppressing the Wnt/β-catenin pathway

**DOI:** 10.18632/oncotarget.2512

**Published:** 2014-09-25

**Authors:** Xin Tong, Lili Li, Xiaoyan Li, Lei Heng, Lan Zhong, Xianwei Su, Rong Rong, Shi Hu, Wenjia Liu, Baoqing Jia, Xing Liu, Geng Kou, Jun Han, Shangjing Guo, Yi Hu, Cheng Li, Qian Tao, Yajun Guo

**Affiliations:** ^1^ International Joint Cancer Institute, The Second Military Medical University, Shanghai, China; ^2^ PLA General Hospital Cancer Center Key Laboratory, Medical School of Chinese PLA, Beijing, China; ^3^ Department of Pharmacy, Liao Cheng University, Shandong, China; ^4^ Cancer Epigenetics Laboratory, Department of Clinical Oncology, State Key Laboratory of Oncology in South China, Sir YK Pao Center for Cancer and Li Ka Shing Institute of Health Sciences, The Chinese University of Hong Kong, Hong Kong; ^5^ 150 hospital of Chinese PLA, Luoyang, China; ^6^ State Key Laboratory of Antibody Medicine & Targeting Therapy and Shanghai Key Laboratory of Cell Engineering & Antibody, Shanghai, China

**Keywords:** SOX10, tumor suppressor gene, digestive cancer, methylation, β-catenin signaling

## Abstract

*SOX10* was identified as a methylated gene in our previous cancer methylome study. Here we further analyzed its epigenetic inactivation, biological functions and related cell signaling in digestive cancers (colorectal, gastric and esophageal cancers) in detail. *SOX10* expression was decreased in multiple digestive cancer cell lines as well as primary tumors due to its promoter methylation. Pharmacologic or genetic demethylation reversed *SOX10* silencing. Ectopic expression of SOX10in *SOX10*-deficient cancer cells inhibits their proliferation, tumorigenicity, and metastatic potentials *in vitro* and *in vivo*. SOX10 also suppressed the epithelial to mesenchymal transition (EMT) and stemness properties of digestive tumor cells. Mechanistically, SOX10 competes with TCF4 to bind β-catenin and transrepresses its downstream target genes via its own DNA-binding property. SOX10 mutations that disrupt the SOX10-β-catenin interaction partially prevented tumor suppression. SOX10is thus a commonly inactivated tumor suppressor that antagonizes Wnt/β-catenin signaling in cancer cells from different digestive tissues.

## INTRODUCTION

The Wnt/Wingless signaling pathway plays important roles in the regulation of various biological processes including cell proliferation/migration/fate during embryogenesis and stem cell development/differentiation. Abnormal activation of Wnt signaling is closely associated with multiple human diseases, including malignancies [1, 2]. During canonical Wnt signaling, the protein levels and activities of its downstream effector-transcriptional coactivator β-catenin is finely regulated. With the help of a protein degradation complex containing scaffold protein Axin, APC, CK1 and GSK3β, cytoplasmic β-catenin is constantly degraded via the ubiquitin-proteasome system when no Wnt ligand is present. However, if Wnt ligands bind to cell surface receptors including Frizzled (Fz) protein and LDL receptor-related protein 6 (LRP6), a signal transduction cascade would be induced to stabilize and activate β-catenin protein. Cytosolic β-catenin will accumulate and translocate into the nucleus, and further interacts with TCF/LEF to bind to their target promoters including *c-Myc*, *CCND1* and *MMP7* via a HMG (High Mobility Group) domain [1, 2].

It has been well documented that SOX (SRY-related- HMG-box) family of proteins are critical transcription factors regulating canonical Wnt/β-catenin signaling in diverse development and disease processes. More than 20 SOX proteins have been identified and classified into seven groups (group A to H) according to their highly conserved HMG sequence similarities. During transcriptional regulation, SOX proteins have to cooperate with specific partner proteins in order to bind to gene promoters in a sequence-specific manner. The human SOX10 is located at 22q13.1 and is highly conserved in vertebrates [7]. SOX10 is expressed in many different cell types and tissues and implicated in neural crest development, nervous system neurogenesis, as well as differentiation of oligodendrocyte, glia and melanocytes [8-11].

Abnormalities (over- or under- expression, or genetic mutations) of SOX factors have been shown to play critical roles in human disease pathogenesis including cancer formation and development. Studies have shown that SOX2, SOX3, SOX4, SOX9 and SOX11 are upregulated and possess oncogenic functions in different types of cancers [12-16], while SOX1, SOX7, SOX11, SOX15 and SOX17 have been identified as tumor suppressors [17-21]. SOX10 was reported to possess tumor-promoting activities in several malignancies including melanoma [22] and gliomas [23]. On the other hand, decreased expression of SOX10 was found to promote tumor cell growth and focal adhesions of Merlin-null schwannoma cells [24]. Therefore, the expression and functional role of SOX10 in cancer development needs more detailed investigation.

We previously identified *SOX10* as a methylated gene in our methylome analysis of digestive cancers [25, 26]. Here, we further analyzed its epigenetic alterations, functions and in-depth mechanisms in digestive cancers including colorectal, gastric and esophageal cancers. We found that SOX10 functions as a tumor suppressor by inducing tumor cell apoptosis, inhibiting invasion, regulating cell EMT and stemness through suppressing Wnt/β-catenin signaling.

## RESULTS

### Epigenetic identification of *SOX10* as a methylated gene

**Figure 1:**
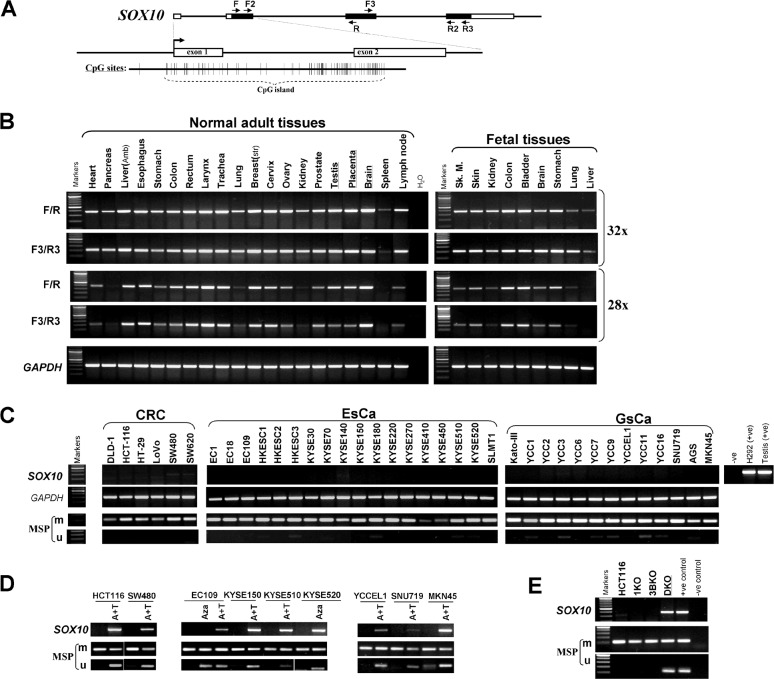
*SOX10* is frequently silenced by promoter CpG methylation in multiple carcinomas (A) Schematic structure of the *SOX10* CpG island. Exon 1,2 (indicated with a black rectangle), CpG sites (short vertical lines). (B) *SOX10* is broadly expressed in human normal adult tissues and fetal tissues. (C) *SOX10* is frequently silenced and methylated in colorectal, esophageal and gastric carcinoma cell lines. Ca, carcinoma; CRC, colorectal cancer; ESCC, esophageal carcinoma; M, methylated; U, unmethylated. (D) Pharmacologic demethylation with Aza alone or combined with TSA (A+T), or (E) genetic demthylation in DKO cell line restored *SOX10* expression in methylated/silenced tumor cell lines.

Semiquantitative RT-PCR showed wide expression of *SOX10* in a series of human normal adult and fetal tissues with variable expression levels, consistent with previous observations [27] (Figure 1A and 1B). In contrast, *SOX10* expression was significantly reduced or completely silenced in multiple digestive tumor cell lines of different histological origins including colorectal, gastric and esophageal cancers, but rarely silenced in melanoma cell lines which acts as a positive control (Figure 1C and Supplementary Figure S1A and S1B). SOX10 was also found to be downregulated in multiple other carcinoma cell lines including nasopharyngeal, lung, and breast (data not shown). The results were further confirmed by two more primer pairs target different regions of *SOX10*. These data indicate that frequent downregulation of *SOX10* is involved in multiple digestive tumorigenesis.

The SOX10 contains a typical CpG island, spanning the promoter, exon 1, intron 1 and part of exon 2 (Figure 1A). We thus further examined *SOX10* promoter methylation by methylation-specific PCR (MSP) and found that *SOX10* was frequently methylated in multiple cell lines, correlated with expression levels (Figure 1C).

To further investigate the relationship between promoter methylation and *SOX10* expression, multiple cancer cell lines with decreased *SOX10* mRNA were treated with DNA-demethylating agent Aza, alone or combined with trichostatin A, a histone deacetylase inhibitor. *SOX10* mRNA was significantly induced in treated cancer cells (Figure 1D). Meanwhile, the *SOX10* promoter was demethylated. Interestingly, the high level of *SOX10* expression in melanoma cell lines is associated with lack of promoter methylation, except for the WM852 cell line (Supplementary FigureS1B). These results demonstrate that promoter methylation mediates transcriptional silencing of *SOX10* in digestive cancers.

We also found that *SOX10* could be activated in the colorectal cancer cell line HCT116 which is completely methylated for this gene, by genetic demethylation through only double knockout (KO) of both DNMT1 and DNMT3B (DKO cell line), but not single KO of DNMT1 or DNMT3B alone (1KO or 3BKO cell line) (Figure 1E). Concomitantly, unmethylated *SOX10* promoter alleles were detected in Aza-treated HCT116 and DKO cells, but not in DNMT1 or DNMT3B single KO cells, indicating that methylation directly mediates *SOX10* silencing. These results also suggest that the maintenance of *SOX10* methylation is mediated by DNMT1 and DNMT3B together.

### *SOX10* expression and methylation in primary digestive tumors

**Figure 2:**
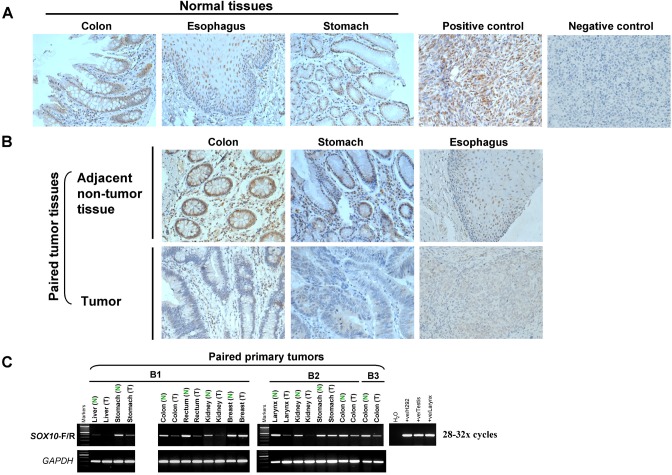
SOX10 expression is significantly decreased in tumor tissues (A) Immunohistochemistry analysis (IHC) was performed with an anti-SOX10 antibody on a human normal tissue microarray (TMA). Melanoma tissue was used as a positive control and nonimmune mouse immunoglobulin G substituted for the primary antibody as negative control (original magnification ×400). (B) SOX10 expression is significantly decreased in different tumor tissues. (C) mRNA expression levels of *SOX10* in different tumor tissues (T) and their paired adjacent normal tissues (N) as determined by semiquantitative RT-PCR (28 or 32 cycles).

We next performed immunohistochemistry (IHC) analyses to examine SOX10 expression in multiple human tissues using a monoclonal antibody described previously [22]. SOX10 expression was first examined in 16 melanomas in a tissue microarray. Clearly, strong nuclear immunostaining of SOX10 was found in all melanoma cases, while the negative control treated identically with a normal mouse IgG antibody showing no staining (Figure 2A), in agreement with previous report [22].

We next analyzed SOX10 expression using a human normal tissue microarray carrying 24 different tissue types. SOX10 was normally expressed in colon, esophagus, stomach, brain, prostate, pancreas, heart and testis and, to a lesser extent, lung tissue, in good conformity with RNA levels (Figure 2A and Supplementary Figure S2A).

SOX10 expression was further detected by IHC in multiple primary carcinomas and paired adjacent non-tumor tissue from the same patients. We found that SOX10 protein was normally expressed in paired non-cancerous colorectal, gastric and esophageal tissues, whereas it was hardly or weakly detected in tumor tissues (Figure 2B). SOX10 expression was decreased or completely silenced in multiple primary digestive cancers with variable frequencies. Downregulation of SOX10 was frequently detected in 51% (20 of 39) of colorectal, 68% (23 of 34) of gastric and 51% (20 of 39) of esophageal cancer tissues.

We further evaluated *SOX10* promoter methylation in primary digestive cancers. Methylation was detected in multiple tumors, including 64% (7/11) of CRC, 77% (40/52) of gastric and 76% (13/17) of ESCC samples (Supplementary Figure S2B). These results demonstrate that *SOX10* silencing by promoter methylation is a frequent event in digestive tumorigenesis.

### SOX10 inhibits digestive tumor cell survival

**Figure 3:**
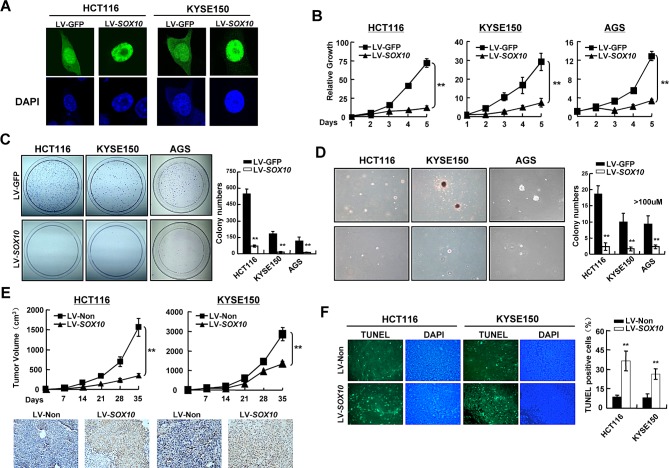
SOX10represses tumor cell survival (A) SOX10 (green) was primarily located in the nucleus in SOX10-transfeced cells by immunostaining. DAPI counterstaining (blue) was used to visualize DNA. (B) Cells were infected with LV-*SOX10* or LV-GFP and seeded in 96-well plates for MTS assay. Values are mean ± SD for triplicate samples from a representative experiment. *: *p*<0.05. **: *p*<0.01. (C) Representative colony formation assays. (D) Representative anchorage-independent growth. (E) Cells infected with LV-*SOX10* or LV-Non were injected subcutaneously into nude mice. Tumor volume of each group was scored every 7 days (Upper). The expresson of SOX10 in the tumors was confirmed by IHC staining (lower). (F) In situ TUNEL apoptosis analysis was performed in tumor sections derived from the same mice as in Figure 3E. The apoptotic nuclei were seen as green color excited under fluorescence microscopy. (magnification ×400).

The observations described above have implicated *SOX10* as a tumor suppressor in digestive cancers. We thus explored whether tumor cells are functionally dependent on SOX10 inactivation. HCT116 (colon), KYSE150 (esophageal) and AGS (gastric) cell lines, all with fully methylated and completely silenced *SOX10,* were infected with lentivirus vectors encoding SOX10 or control GFP. SOX10 proteins were found localized predominantly in the nucleus (Figure 3A). Ectopic expression of SOX10 in these cells significantly suppressed cell growth, colony formation and anchorage-independent growth (Figure 3B-3D). To further confirmed the inhibitory effects of *SOX10* on tumor growth *in vivo*, HCT116 and KYSE150 cells infected with LV-*SOX10* or LV-GFP were implanted subcutaneously into the flank of nude mice. Compared to LV-GFP control group, expression of SOX10 significantly inhibited tumor growth in nude mice (Figure 3E). Together, these data demonstrate that SOX10 functions as a tumor suppressor for digestive cancers.

The inhibitory effects of SOX10 on tumor growth were likely the result of apoptosis induction as revealed by TUNEL assay of HCT116 and KYSE150 xenografted tumor tissues. When elevating the level of SOX10, the apoptotic nuclei, seen as green color excited under fluorescence microscope, were greatly increased (Figure 3F).

### SOX10 represses tumor cell metastasis

**Figure 4:**
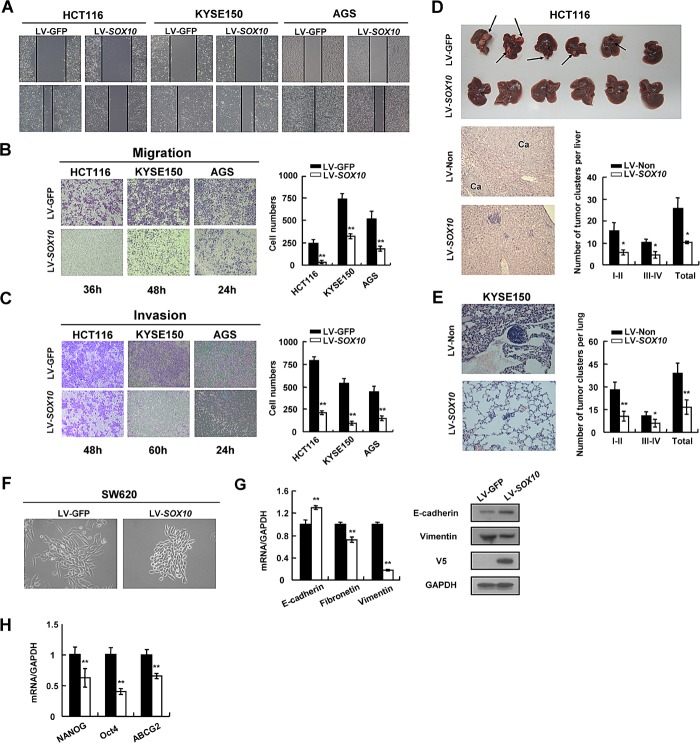
SOX10 suppresses tumor metastasis and EMT phenotype Cells were infected with LV-*SOX10* or LV-GFP and subjected to wound-healing assay (A), migration assay (B) and invasion assay (C). Microscopic observations were recorded at 0 as well as 48 or 72 hours after scratching the surface of a confluent layer of cells(A). Cells that migrated (B) or invaded (C) to the lower chamber were fixed, stained, and counted using light microscopy. Values are mean ± SD for triplicate samples from a representative experiment. *: *p*<0.05. **: *p*<0.01. (D) HCT116 cells infected with LV-*SOX10* or LV-GFP were inoculated into the spleen. The mice were killed and examined for the presence of hepatic metastasis 12 weeks after the intrasplenic inoculation. The upper, metastatic nodules on the surface of the liver are shown; the left, representative H&E staining of liver (original magnification, ×400); the right, the numbers of nodules were quantified and values for each group are denoted (*: *p*<0.05). (E) Male Balb/c nude mice were injected subcutaneously with 5×10^6^ KYSE150 cells. Representative lung tissue sections by HE-staining from each group were shown in the left (magnification ×400). The number of lung metastatic foci in each group was calculated under microscope. (F) Morphology changes of SW620 cells infected with LV-*SOX10* or empty vector by phase contrast microscopy. Original magnification, ×400. (G) qRT-PCR and western blot validation of EMT biomarkers (*: *p*<0.05, **: *p*<0.01). GAPDH was used as a control. (H) Downregulation of representative stem cell markers in SOX10-infected tumor cells.

The effect of SOX10 on tumor cell migration and invasion was further explored. HCT116, KYSE150 and AGS cells infected with LV-*SOX10* filled the wound much slower than LV-*GFP* infected cells (Figure 4A). Induction of SOX10 also markedly inhibited cell migration through a permeable filter and invasion through Matrigel (Figure 4B and 4C). Subsequently, we performed two sets of metastasis assays in nude mice. As shown in Figure 4D, lung metastatic clusters presented in mice subcutaneously injected with KYSE150-SOX10 cells (16.6±4.8 clusters per mice) were significantly fewer than those in the LV-*GFP* group (38.8±6.8 clusters per mice), as examined by HE staining. Notably, overexpression of SOX10 suppressed the establishment of colorectal cancer liver metastasis (Figure 4E). Collectively, these observations indicate that SOX10 negatively regulates metastatic properties of digestive cancer cells.

### SOX10 suppresses the EMT phenotype and stem cell property of tumor cells

Given that SOX10 inhibits cancer metastasis, we wonder whether SOX10 plays a role in regulating epithelial-mesenchymal transition (EMT), a critical event in tumor invasion [28, 29]. To test this hypothesis, we selected the SW620 colon cancer cell line which is derived from a lymph node metastasis of a colorectal tumor. SW620 cells infected with LV-GFP showed a prominent mesenchymal-like phenotype, including a fibroblast-like morphology, loss of cell-cell contacts, expression of the mesenchymal marker vimentin as described previously [30, 31]. However, ectopic expression of SOX10 in SW620 cells led to cobblestone morphology in monolayer cultures, with tight cell-cell contacts characteristics of normal epithelial cells (Figure 4F), indicating that SOX10 most likely reversed tumor cell EMT. This was accompanied by an increase of epithelial cell marker, such as E-cadherin, a decrease of mesenchymal cell markers, such as fibronectin and vimentin and an increased migration ability (Figure 4G and Supplementary Figure S3A). Furthermore, SOX10 expression downregulated 3 stem cell markers: *NANOG*, *ABCG2* and *Oct4*, at the transcriptional level (Figure 4H). Taken together, these results suggest that SOX10 inhibits both the EMT and stemness of digestive cancer cells.

### SOX10 inhibits the β-catenin signaling pathway in digestive tumor cells

**Figure 5:**
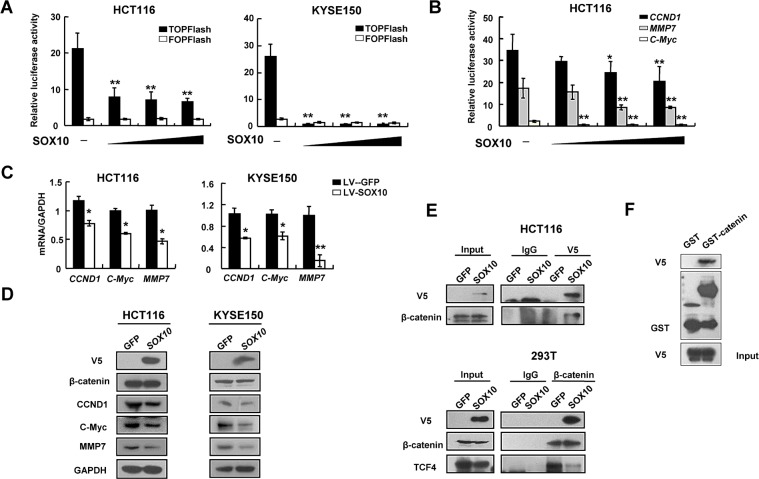
SOX10 suppresses Wnt signaling via compete with TCF4 to bind β-catenin Inhibition of TCF activity (A) and *CCND1*, *c-Myc* and *MMP7* promoter reporter activities (B) in SOX10-expressing tumor cells in a dose-dependent manner. *: *p*<0.05, **: *p*<0.01. qRT-PCR (C) and western blot (D) showed ectopic expression of SOX10 disrupted Wnt signaling pathway. (E) Co-immunoprecipitation (Co-IP) assays showing that exogenous V5-SOX10 binds endogenous β-catenin and vice versa. IP antibody or IgG negative control was used as indicated. (F) GST pull-down assay was performed with GST-β-catenin fusion protein or GST and V5-SOX10 as indicated.

Previous studies have shown that SOX family members play important roles in modulating Wnt/β-catenin pathway in diverse development and disease contexts [3]. To examine whether the above observed SOX10-dependent effects were mediated by β-catenin, we performed reporter assays using both the TOPFlash construct harboring multiple TCF/LEF-binding sites and the derived FOPFlash construct with mutated TCF/LEF-binding sites as a negative control. Overexpression of SOX10 repressed the TOPFlash activity in a dose-dependent manner in KYSE150 cells, while they had no effect on the FOPFlash control (Figure 5A). The impact of SOX10 overexpression on β-catenin/TCF activity was further examined in HCT116 colon cancer cells which contain a heterozygous activating mutation in β-catenin [20]. SOX10 inhibits β-catenin promoter activities in a dose-dependent manner (Figure 5A). Similarly, increasing the amount of exogenous SOX10 progressively repressed the activities of *CCND1*, *c-Myc* and *MMP7* promoter constructs as well as their expression in HCT116 cells (Figure 5B and 5C), which are typical transcriptional targets of the Wnt/β-catenin/TCF signaling pathway. Collectively, these data suggest that SOX10 can antagonize the canonical Wnt/β-catenin signaling cascade in digestive cancer cells.

### SOX10 competes with TCF4 for interacting with β-catenin

SOX proteins regulate β-catenin/TCF activity through different mechanisms including protein-protein interactions [3]. To test whether SOX10 could interact with β-catenin, we performed co-immunoprecipitation (co-IP) analyses using cell lysates from HCT116 cells transfected with V5-tagged SOX10, and found that endogenous β-catenin could bind to SOX10 and vice versa (Figure 5D and 5E). Consistent with the findings from *in vitro* binding assays, V5-tagged SOX10 was able to pull down GST-tagged β-catenin (GST-β-catenin) but not GST alone, suggesting that SOX10 directly interact with β-catenin (Figure 5F).

In order to regulate gene expression, β-catenin has to interact with TCF4, then is recruited to target promoters via TCF4's HMG domain. As SOX10 also contains a HMG domain, we tried to determine whether SOX10 and TCF4 were present in the same complex with β-catenin or compete with each other for β-catenin binding. Using anti-β-catenin antibody to pull down endogenous β-catenin in 293T cells, we detected endogenous TCF4 coprecipitated with β-catenin in the GFP group, but virtually not in the SOX10-overexpressed group, indicating that SOX10 is directly competing with TCF4 for β-catenin binding (Figure 5E).

### Interaction of SOX10 with β-catenin and its DNA binding ability are required for tumor suppression

**Figure 6:**
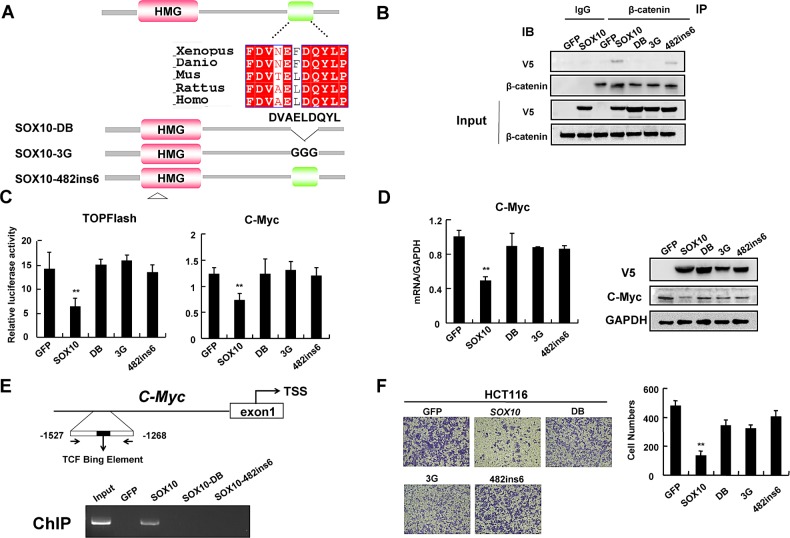
Interaction of SOX10 with β-catenin and its DNA binding ability are required for tumor suppression (A) The schematic shows that the SOX10 contains a candidate β-catenin-binding region which is highly conserved. Below the schematic the ‘DB’, ‘3G’ and ‘482ins6′ mutations generated in SOX10 are shown. (B) IP assays showing that endogenous β-catenin binds exogenous V5-SOX10 and 482ins6 mutants, but not DB or 3G mutants. (C) HCT116 cells were cotransfected with V5-SOX10 or mutants plus the TOPFlash or *c-Myc* promoter reporter. Values are mean ± SD for triplicate samples from a representative experiment. *: *p*<0.05, **: *p*<0.01. (D) qRT-PCR and western blot showed ectopic expression of GFP, SOX10 and mutants on Wnt/β-catenin signaling pathway. (E) Schematic structure of the *c-Myc* promoter. TCF Binding Element (TBE) are indicated (Upper). ChIP assays on Wnt-responsive elements of *c-Myc* promoter gene were performed in HCT116 cells that were transfected with GFP, V5-SOX10 and mutants (lower). (F) Migration assay testing the ability of HCT116 cells expressing GFP, SOX10 and mutants to migrate through transwell chambers.

Many SOX proteins have been shown to physically interact with β-catenin through a short 9 amino acid motif (DxxEFDQYL), which shows a high degree of similarity to a loosely conserved armadillo-binding sequence found in many β-catenin-binding proteins [3, 32]. Analysis of the amino acid sequence of SOX10 revealed a match with the motif DVAELDQYL which is evolutionarily conserved (Figure 6A). To test if this conserved motif is essential for protein interaction, we mutated the conserved amino acids DQY to GGG (referred to as 3G mutant) or deleted amino acids ELDQY (referred to as DB mutant) in SOX10 (Figure 6A). Co-IP showed that endogenous β-catenin binds to V5-tagged wild-type SOX10, but not 3G or DB mutant (Figure 6B), suggesting that DVAELDQYL motif is indispensable for direct β-catenin binding.

SOX10 is a transcription factor binding DNA sequence via a HMG domain. As our above data indicated that SOX10 competed with TCF4 to bind β-catenin, we next investigated whether its DNA-binding activity was required to antagonize β-catenin/TCF activity. We generated the SOX10-482ins6 mutant, which carries an insertion of 6 nucleotides between positions 482 and 483, resulting in the addition of a leucine and an arginine residue into the HMG box and disruption of the structure of SOX10 DNA-binding domain [33] (Figure 6A). This mutant maintained the same ability to interact with β-catenin but failed to repress the β-catenin/TCF reporter (Figure 6B and 6C), indicating that the repressive effect of SOX10 on β-catenin/TCF transcriptional activity depends on its DNA-binding property.

To further confirm that SOX10 was recruited to Wnt/β-catenin target gene promoters and transrepressed their expression via HMG domain instead of interacting with β-catenin and sequestering it away from target promoters, we performed a ChIP assay on *c-Myc* promoter. As expected, ChIP analysis demonstrated that SOX10 was recruited to *c-Myc* promoter in HCT116 cells, while the SOX10-DB mutant or SOX10-482ins6 failed to bind to *c-Myc* promoter (Figure 6E), indicating that SOX10-mediated repression of Wnt/β-catenin target genes depends on its interaction with β-catenin as well as its own DNA-binding property.

Cumulative outcomes from our above studies supported a key role of β-catenin inhibition in SOX10-induced tumor suppression of digestive tumor cells. Therefore, we next questioned whether the capacity to bind β-catenin is instrumental for SOX10 function. We first examined whether SOX10 DB or 3G mutants lose their ability to suppress Wnt/β-catenin signaling. Indeed, while ectopic SOX10 expression in HCT116 cells significantly inhibited the TOPFlash reporter as compared to control cells, transfection with SOX10-DB or SOX10-3G mutants largely reversed the suppressive effect on the reporter (Figure 6C). qRT-PCR and western blot analysis confirmed that mutations or deletion of the conserved DVAELDQYL motif as well as HMG domain mutation impaired the ability of SOX10 to attenuate Wnt signaling (Figure 6D). Furthermore, tumor cells with ectopic SOX10 expression migrated significantly slower than control cells, whereas introduction of SOX10-DB, -3G or -482ins6 mutants only partially suppressed tumor cell migration as by transwell assays (Figure 6F). These results further suggest that the interaction of SOX10 with β-catenin as well as its DNA binding ability are, at least in part, required for its tumor suppressor functions.

## DISCUSSION

In this study, we found that while SOX10 is ubiquitously expressed in human normal adult and fetal tissues, it is frequently silenced or downregulated due to promoter methylation in digestive (colon, gastric and esophageal) cancer cell lines and primary tumors. We found that ectopic expression of SOX10 suppressed tumor cell proliferation and tumorigenicity through promoting apoptosis *in vitro* and in nude mice. Furthermore, we found that SOX10 inhibited the EMT and stemness of tumor cells and their migration and invasion through antagonizing Wnt/β-catenin signaling. Our study thus validated that SOX10 is a functional tumor suppressor for digestive cancers.

The canonical Wnt signaling pathway regulates multiple fundamental cellular processes including cell proliferation, migration and stemness as well as tumorigenesis, largely through modulating target gene transcription [1, 2]. Mutations in Wnt pathway components such as APC or stabilizing mutations in β-catenin itself that lead to constitutive signaling are frequently found in digestive cancers [34, 35]. Epigenetic downregulation of tumor suppressor genes, especially in the setting of key signaling pathways, have been proven to be a complementary mechanism to genetic mutation during carcinogenesis [36]. Some important Wnt signaling inhibitors such as *SFRPs* [37], *WIF1* [38], *DKKs* [39] as well as *SOX1*, *SOX7,SOX15* and *SOX17* have been previously reported to be frequently methylated in multiple tumors. We reported here that *SOX10*, another SOX family member, functions as a TSG but is silenced by promoter CpG methylation in multiple digestive cancers. In melanomas, upregulation of SOX10 correlates well with promoter hypomethylation. These data suggest promoter methylation could be the major mechanism determining whether SOX10 is silenced or activated in tumors.

SOX familiy members regulate canonical Wnt signaling through different mechanisms, including protein-protein interactions, binding of SOX factors to Wnt-target gene promoters, the recruitment of co-repressors or co-activators, modulation of protein stability, and nuclear translocation [3]. Many SOX proteins can physically interact with β-catenin via a conserved 9 amino acid motif (DXXEFDQYL), for example, SOX7 and SOX17 [3]. Sequence analysis indicate that SOX10 also contains a similar highly conserved motif, DVAELDQYL, which is essential to the intercation of SOX10 with β-catenin, also to the abilities of SOX10 to bind to target promoters and exert its transcriptional regulator and tumor suppressor functions.

We used digestive cancer cell lines with mutant (HCT116 [40] and AGS [41] ) or wild-type β-catenin (KYSE150), all of which have complete silenced expression of SOX10. Ectopic expression of SOX10 could effectively suppress tumor proliferation and metastasis in cell lines harboring wild-type as well as mutant β-catenin. Consistent with this, SOX10 could interact with mutant β-catenin and repress target gene transcription. These data suggest that SOX10 functions as a tumor suppressor probably independent of β-catenin mutation and is another checkpoint for aberrant β-catenin activation in cancers.

As no DNA binding domain existed in β-catenin, other DNA binding partners are needed to assist β-catenin to its target promoters. Here, we discovered that SOX10 could compete with TCF4 to form a complex with β-catenin and be recruited to target promoters to suppress their expression. This transrepresson ability is dependent on the DNA-binding activity of SOX10's HMG domain, because a SOX10 mutant that is incapable of DNA binding can still interact with β-catenin, but fails to suppress the TOPFlash reporter as well as Wnt-target gene expression. Together, these findings demonstrated the unexpected role of SOX10 as an important DNA binding transcription factor that recruits β-catenin to repress Wnt/β-catenin signaling in cancer progression.

Since disrupting SOX10-β-catenin interaction could only partially abolished tumor suppressor functions of SOX10, there may be other signaling pathways participated in the complex mechanisms of tumor suppression by SOX10. This issue remains to be explored in further depth in future.

Although emerging evidences together with ours support that SOX10 functions as a tumor suppressor in certain tumors, overexpression and oncogenic property of SOX10 have been reported in melanoma and glioma, indicating that SOX10 plays a complex role in tumorigenesis. The SOX family of transcription factors regulate a wide range of biological events by cooperating with specific partner factors to select specific target genes. When a SOX-partner is changed, the set of activated genes might be dramatically altered. For example, during Schwann cell development, SOX10 cooperates with OCT6/BRN2 to upregulate the *Krox20* gene. However, during melanocyte development, SOX10 is partnered with PAX3 and activates the gene encoding *MITF* [5, 42]. In melanoma, SOX10 haploinsufficiency abrogates *Nras*^Q61K^-driven tumor initiation as well as progression [22]. SOX10 increases KROX20 and myelin protein zero (MPZ) expression, and promotes NFATC4 nucleus translocation, further suppressing Merlin-null cell proliferation [24]. These results suggested that SOX10 function in a context- and likely cell-type dependent manner. The molecular mechanisms and biological functions of SOX10 in other tumors need to be determined in future studies.

In summary, our study identifies SOX10 as a functional tumor suppressor and an important regulator of the Wnt/β-catenin signaling pathway, with frequent epigenetic inactivation in certain digestive tumor. These findings improves our understanding of the molecular mechanisms underlying β-catenin activation and tumor progression and provide a potential target for cancer therapy.

## MATERIALS AND METHODS

### Cell lines and culture condition

Tumor cell lines were obtained from American Type Culture Collection. Colon cancer cell line HCT116 with genetic knock-out (KO) of DNA methyltransferases (DNMT): HCT116 DNMT1^−/−^ (1KO), HCT116 DNMT3B^−/−^ (3BKO) and HCT116 DNMT1^−/−^ DNMT3B^−/−^ (DKO) cells (gifts of Bert Vogelstein, Johns Hopkins University Medical Institutes) were grown with either 0.4 mg/ml genecitin or 0.05 mg/ml hygromycin or both.

### Tissue samples and immunohistochemistry (IHC)

Normal adult and fetal tissue RNA samples were purchased commercially (Stratagene, La Jolla, CA, USA or Millipore-Chemicon, Billerica, MA,). DNA samples of primary carcinomas (T) and matched surgical margin normal tissues (N) have been described previously. For IHC staning, human normal tissue TMA composed of 24 different tissue types (Superchip Co., Ltd., Shanghai, China) and Human melanoma tissue TMA (US Biomax, Rockville, MD) was utilized. Paired tumor tissues were collected in Luoyang 150 Hospital and Chinese PLA General Hospital in China. Tissue samples were collected with informed consent and the procedure was approved by the hospital ethics committees. Further details can be found in the Supplementary information.

### DNA bisulfite treatment, methylation analysis, 5-Aza-2′-deoxycytidine(Aza) and trichostatin A treatment

See Supplementary information.

### Plasmid construction and lentivirus production

See Supplementary information.

### qRT-PCR, semi-quantitative RT–PCR, western blot and immunofluorescence staining, dual-luciferase reporter assay

See Supplementary information.

### MTS Assay, colony formation assay, anchorage-independent growth assay, TUNEL Assay, wound-Healing, transwell migration and invasion assay

See Supplementary information.

### Glutathione-S-transferase pull-Down assay

Glutathione-S-transferase (GST) and GST-tagged β-catenin were expressed in Escherichia coli and purified by glutathione-sepharose beads (GE Healthcare). We incubated SOX10 protein extracted from 293T cells transfected with pcSOX10 with GST or the GST-catenin for 1 hour at 4°C. After washing, we resolved the adsorbed proteins by sodium dodecyl sulfate (SDS)-polyacrylamide gel electrophoresis and analyzed them by western blotting. Further details are available in the Supplementary information.

### Co-immunoprecipitation (IP)

Whole-cell extracts of HCT116 or 293T cells expressing V5-SOX10 or its mutants were immunoprecipitated with anti-V5 or anti-β-catenin, followed by western blot analysis using anti-V5, anti-β-catenin, or anti-TCF4 antibodies. Further details are available in the Supplementary information.

### ChIP assay

ChIP was performed using the EZ ChIP™ Chromatin Immunoprecipitation Kit (Millipore, Billerica, MA) according to the manufacturer's protocol. Sonicated chromatin was immunoprecipitated with anti-V5 antibody and normal mouse IgG as the negative controls. Immunoprecipitated DNA was then reverse cross-linked, purified and subjected to PCR. Primers used in this study are listed in Supplementary information, Table S1.

### Animal studies

Male BALB/c nude mice at six week-old were housed under standard conditions and cared for according to the institutional guidelines for animal care. All animal experiments were approved by Institutional Animal Care and Use Committee (IACUC) of PLA General Hospital. Further details can be found in the Supplementary information.

### Statistic analysis

The analyses were carried out using SPSS 16.0 for Windows software (SPSS, Chicago, IL, USA). Results are shown as values of mean ± SD. Statistical analyses were performed using Student's t test to determine *P* values.

## SUPPLEMENTAL MATERIAL TABLE AND FIGURES



## Abbreviations

APC: adenomatous polyposis coli
ChIP: chromatin-immunoprecipitation
CK1: casein kinase 1
CRC: colorectal cancer
EMT: epithelial-mesenchymal transition
DAPI: 4′,6-diamidino-2-phenylindol
ESCC: esophageal cancer
GSK3β: glycogen synthase kinase 3β
GST: Glutathione-S-transferase
IF: Immunofluorescence
IHC: Immunohistochemisty
IP: Immunoprecipitation
qRT-PCR: quantitative reverse-transcription polymerase chain reaction
TCF/LEF: T cell factor/lymphoid enhancer factors
TMA: tissue microarray
TSG: tumor suppressor gene
TUNEL: terminal deoxynucleotidyl transferase dUTP nick end labeling.
