# Supplementary material for: SOX10, a novel HMG-box-containing tumor suppressor, inhibits growth and metastasis of digestive cancers by suppressing the Wnt/β-catenin pathway
Source: Oncotarget. 2014 Sep 25;5(21):10571–83. doi: 10.18632/oncotarget.2512 (PMC4279394; doi:10.18632/oncotarget.2512)
Supplement: Supplementary file 1 [file oncotarget-05-10571-s001.pdf]

SOX10, a novel HMG-box-containing tumor suppressor, inhibits growth and metastasis of digestive cancers by suppressing the Wnt/β-catenin pathway

Supplemental Material

Materials and Methods

Plasmid construction

V5-tagged SOX10 was constructed by cloning full-length cDNA into pLVX-AcGFP-N1 vectors (Clontech, Mountain View, CA), designated as LV-*SOX10*, with empty vector as negative control (designated as LV-GFP or LV-Non). The DB, 3G and 482ins6 mutation constructs were generated with site-directed mutagenesis (Stratagene, La Jolla, CA). GST-tagged β-catenin mutants were constructed by cloning β-catenin (138-686) into PGEX-4T2 vector (GE Healthcare Life Sciences). The TOPFlash reporter and FOPFlash reporter were kindly provided by Prof. Christof Niehrs (German Cancer Research Center, DKFZ). *CCND1*-, *c-Myc*- and *MMP7*- promoter reporter constructs containing TCF-responsive element were cloned in our Lab. All constructs were confirmed by DNA sequencing.

Lentivirus production and infection

To produce lentivirus containing SOX10 gene, HEK-293T cells were cotransfected with the resulting vector described above and ViraPower Packaging Mix (Life Technologies, Carlsbad, CA) with the use of Lipofectamine 2000 according to the manufacturer's guidelines. Infectious lentiviruses were harvested at 48 and 72 hours after transfection and filtered through 0.45-μm polyvinylidene diflouride filters (Millipore, Bedford, MA). Recombinant lentiviruses were concentrated 100-fold by ultracentrifugation (2 hours at 50,000g). The

virus-containing pellet was dissolved in DMEM, divided into aliquots, and stored at −80°C. The infectious titer was determined by counting the blue-stained colonies after crystal violet staining in 293T cells.

Cells were infected with concentrated virus at a multiplicity of infection of 20 in the presence of 8 μg/mL polybrene (Sigma-Aldrich, St. Louis, MO). Supernatant was removed after 24 hours and replaced with complete culture medium. Ninety-six hours after infection, the transduced cells were confirmed by western blot.

Antibodies

Antibodies used were: E-cadherin (4065), TCF4 (2569), Vimentin(3932), β-catenin(9562) (Cell Signaling, Beverly, MA). Anti-mouse IgG-HRP (P0161), anti-rabbit IgG-HRP (P0448) (Dako, Glostrup, Denmark). GST (clone 56C1, sc-80998), CCND1 (sc-20044), c-Myc (sc-764), (Santa Cruz, CA, USA). MMP7 (MS-813-P0, Thermo Scientific, Fremont, CA). GAPDH-HRP (Kangchen Biotechnology). Monoclonal SOX10 antibody (clone 20B7, MAB2864, R&D Systems). Anti-V5 tag antibody (clone SV5-Pk1, MCA1360GA, AbD Serotec). β-catenin (clone 14, 610154, BD Biosciences, SanJose, CA.)

Semiquantitative reverse transcription-PCR and qRT-PCR

Total RNA was isolated with the NucleoSpin RNA II Kit (MACHEREY-NAGEL, Düren, Germany) with genomic DNA removed by RNase-Free DNase included. Reverse transcription using random hexamer and reverse transcription-PCR using Go-Taq (Promega, Madison, WI) were done as previously with GAPDH as an endogenous control. Reverse transcription-PCR was done for 28 or 32 cycles for SOX10, but only 23 cycles for GAPDH. Quantitative RT-PCR was performed with ABI 7500 system (Applied Biosystems, Darmstadt,

Germany). Relative expression of genes was calculated and expressed as $2^{-\Delta\Delta CT}$. The primers used in this study are listed in the Supplementary information, Table S1.

DNA Bisulfite treatment and methylation analysis

DNA bisulfite treatment, MSP, and BGS were carried out as described[1, 2]. MSP was done for 40 cycles using AmpliTaq Gold and hot start. MSP primers were tested previously for not amplifying any unbisulfited DNA and the specificity of MSP was further confirmed by direct sequencing of some PCR products. The PCR products were subcloned into the pGEM-Teasy vector (Promega), with 6 to 8 colonies randomly chosen and sequenced. The primers used in this study are listed in the Supplementary information, Table S1.

5-Aza-2'-deoxycytidine and Trichostatin A Treatment

Tumor cells were allowed to grow overnight. The culture medium was then replaced with fresh medium containing 5-aza-2′-deoxycytidine (Aza) at a final concentration of 5 to 10 μmol/L (Sigma-Aldrich).Cells were allowed to grow for 72 hours with change of Aza-containing medium every 24 hours; some were further treated with the HDAC inhibitor trichostatin A (TSA) for an additional 24 hours. Cells were then harvested for DNA and RNA extractions.

Tissue Samples

For IHC, tissue samples as well as corresponding adjacent non-tumor tissues were obtained from Chinese PLA General Hospital and Luoyang 150 Hospital. The use of all the human samples and the experimental procedures for this study were reviewed and approved by the hospital ethics committees. Informed consent was obtained from all subjects included. The specimens were snap-frozen in liquid nitrogen and stored at -80°C for molecular analyses.

The remaining tissue specimens were fixed in 10% formalin and embedded in paraffin for routine histologic examination. All the paraffin-embedded tissue specimens were diagnosed and reconfirmed by two experienced pathologists.

Immunohistochemistry and Scoring

The expressions of SOX10 protein in the specimens were detected by Dako REAL™ EnVision™ Detection System (DAKO) as described previously [3]. Briefly, sections were cut at 4μm and dried at 60°C. The sections were deparaffinized in xylene, hydrated through graded alcohols and rinsed in distilled water. Heat-induced epitope retrieval was performed in a pressure cooker in 10 mM citrate buffer (pH 6.0) for 5 min. Sections were cooled for 30 min and then were rinsed in PBS. Endogenous peroxidase activity was blocked by hydrogen peroxide treatment. The antibody against SOX10 was diluted at 1:100 and incubated overnight at 4°C. Secondary detection was accomplished using horseradish peroxidase-labelled secondary anti-mouse antibody and staining was visualized using the peroxide substrate solution diaminobenzidine followed by light nuclear counterstaining with hematoxylin. For antibody control, one set of samples was incubated with non-immune mouse IgG (1:100) instead of primary antibody as negative control and four melanoma tissue specimens as positive control.

Evaluation of SOX10 staining was independently performed by two experienced pathologists. The intensity of SOX10 immunostaining was semiquantitatively estimated according to the signal intensity and distribution. Briefly, a mean percentage of positive tumor cells was determined in at least five areas ×400 magnification and assigned to one of the five following categories: 0, <5%; 1, 5-25%; 2, 25-50%; 3, 50-75% and 4, >75%. The intensity of

immunostaining was scored as follows: 1, weak; 2, moderate and 3, intense. The percentage of positive tumor cells and the staining intensity were multiplied to produce a weighted score for each case. Tissues with immunohistochemical scoring ≤2 were considered as negative, 3-12 as positive.

Western blot

Total cell lysate was prepared in 1×SDS lysis buffer (62.5 mM Tris-HCl, 2% w/v SDS, 10% glycerol, 50 mM DTT, 0.01% w/v bromophenol blue). Proteins at the same amount were separated by 10% SDS-PAGE and transferred onto PVDF membranes. After probing with antibodies, the signals were visualized by Immobilon™ Western Chemiluminescence HRP Substrate (Millipore Corporation).

MTS assay

Cell growth was measured by 3-(4,5-dimethyl-thiazol-2yl)-5-(3-carboxymethoxyphenyl)-2-(4-sulfophenyl)-2H-tetrazolium (MTS) assay (Promega) in 96-well plates (1,000 cells per well) following the instructions of the manufacturer.

Colony formation assay and anchorage-independent growth assay

For colony formation assay, cells were cultured overnight in a 10 cm plate (1.0×10^4 per well) and infected with LV-GFP or LV-*SOX10*. After 14 days, surviving colonies were stained with gentian violet after methanol fixation and visible colonies (≥50 cells) were counted.

For anchorage-independent growth assay, the cells in single-cell suspension were plated in 0.3% agarose over a 0.7% agarose bottom layer at a density of 1.0×10^4 cells per well in 6-well plates and incubated for 14 days and stained. Colonies with a diameter greater than

100 μm were counted. Each experiment was done in triplicate and repeated for three times.

Wound-healing assay

For wound-healing assay, the cells were first seeded in 6-well culture plates. A wound was made in the confluent monolayer with a plastic pipette tip and the migration of the cells at the wound front was photographed using an inverted microscope at indicated times after the scratch.

Transwell migration and invasion assay

Transwell migration assays were quantified in vitro using Transwell chambers with polycarbonate membrane filters (8 μm pore size; Corning) according to the manufacturer's instructions. In brief, the lower chamber was filled with 0.6 ml medium containing 20% fetal bovine serum, and 0.2ml of medium that contained 1×10^5 cells under serum-starving conditions was plated in the upper chamber and incubated at 37°C. Then cells that had not migrated were removed from the upper face of filters using cotton swabs. The cells that migrated through the membrane and attached to the bottom of the membrane were fixed and stained with crystal violet. Images of five random fields were captured from each membrane and the number of migratory cells was counted, and the extent of migration was expressed as the average number of cells per microscopic field at a magnification of 100. All experiments were performed for three times. Similar inserts coated with Matrigel (BD Biosciences) were used to determine invasive potential in invasion assay. Two independent investigators were blinded when reading the migration and invasion assays.

Immunofluorescence staining

Cells were grown on glass chamber slides fixed with 4% paraformaldehyde,

permeabilized with 0.2% Triton X-100/PBS, and blocked with 10% BSA in PBS. Cells were incubated with primary antibody against V5 and then incubated with FITC-conjugated (F0313, Dako) secondary antibody against mouse IgG. Slides were then counterstained with 4′,6-diamidino-2-phenylindol (DAPI) and imaged with an Olympus BX51 microscope (Olympus Corporation, Tokyo, Japan).

Dual-Luciferase reporter assay

Cells were transiently transfected with luciferase reporters as indicated and Renilla vector (pRL-TK) in triplicate using ScreenFect® A Transfection Reagent according to the manufacturer’s instructions (Incella GmbH, Germany). After 48 hours, cells lysated and analyzed with the Dual Luciferase Reporter Assay System (Promega) on a Centro XS 3 LB 960 (Berthold Technology GmbH & Co KG, Germany).

Animal experiments

HCT116 and KYSE150 cells were infected with LV-*SOX10* or LV-Non at a MOI of 20. Viability of cells was determined by trypan blue exclusion staining 4 days after infection and viable cells ($5×10^6$) were injected subcutaneously into the right flank of each mouse (6 mice/group). The tumor volume was calculated using the equation V (mm^3) = $a×b^2/2$, where *a* is the largest dimension and *b* is the perpendicular diameter. Animals were killed for histopathology examination at day 35 after cell inoculation. The tumors and lungs were excised and embedded in paraffin. Sections (4 µm) of tumors and lungs were stained with H&E to visualize the tumor structure or pulmonary metastasis under microscope.

For liver metastasis assay, $5×10^6$ cells of HCT116 infected with LV-*SOX10* or LV-Non

were injected into the spleens of BALB/c nude mice (8 week old; n=6) under anesthesia. The spleens were removed after 10min tumor cell injection to prevent splenic tumor formation. Nude mice were sacrificed at 8 weeks, and the number and size of metastatic tumor foci on the surface of the livers were documented. The livers were excised and embedded in paraffin for histopathology examination.

Supplementary Table S1: Sequences of primers used in the study

PCR	Primers	Sequence (5’-3’)
RT-PCR	SOX10F	AGCTGGGCAAGGTCAAGAAG
	SOX10R	TGGGCTGGTACTTGTAGTCC
	SOX10F2	TGAACGCCTTCATGGTGT
	SOX10R2	CGACTGCAGCTCTGTCTT
	SOX10F3	CACTACAAGAGCGCCCACT
	SOX10R3	TGCCGAAGTCGATGTGAGG
	GAPDHF	GATGACCTTGCCCACAGCCT
	GAPDHR	ATCTCTGCCCCCTCTGCTGA
qRT-PCR	E-cadherinF	TGCCCAGAAAATGAAAAAGG
	E-cadherinR	GTGTATGTGGCAATGCGTTC
	VimentinF	GAGAACTTTGCCGTTGAAGC
	VimentinR	GCTTCCTGTAGGTGGCAATC
	FibronectinF	CAGTGGGAGACCTCGAGAAG
	FibronectinR	TCCCTCGGAACATCAGAAAC
	ABCG2F	CAGTGTCACAAGGAAACACC
	ABCG2R	GAGACCAGGTTTCATGATCC
	NanogF	GATTTGTGGGCCTGAAGAAA
	NanogR	TTGGGACTGGTGGAAGAATC
	Oct4F	CTTGCTGCAGAAGTGGGTGGAGGAA
	Oct4R	CTGCAGTGTGGGTTTCGGGCA
	CCND1F	AACTACCTGGACCGCTTCCT
	CCND1R	CCACTTGAG CTTGTTCACCA
	C-MycF	GCCCCTCAACGTTAGCTTCA
	C-MycR	TTCCAGATATCCTCGCTGGG
	MMP7-F	GAGTGAGCTACAGTGGGAACA
	MMP7-R	CTATGACGCGGGAGTTTAACAT
MSP	SOX10m5c	TTTTTTTTTTTTAGTTTTTCGTAGC
	SOX10m6	ACCTCAAACCACGACCTCG
	SOX10u5	TTTTTTTTTTTTTAGTTTTTTGTAGT
	SOX10u6	AACCTCAAACCACAACCTCA
	SOX10bm1	CGCCCTCGAAACTACGCG
	SOX10bm2	GTTTTTTTCGGGTTAGTCGTC
	SOX10bu1N	GGACCACCCTCAAAACTACACA
	SOX10bu2N	GGTTGTTTTTTTTGGGTTAGTTGTTG
ChIP	C-Myc-Promoter-F	GTGAATACACGTTTGCGGGTTAC
	C-Myc-Promoter-R	AGAGACCCTTGTGAAAAAAACCG

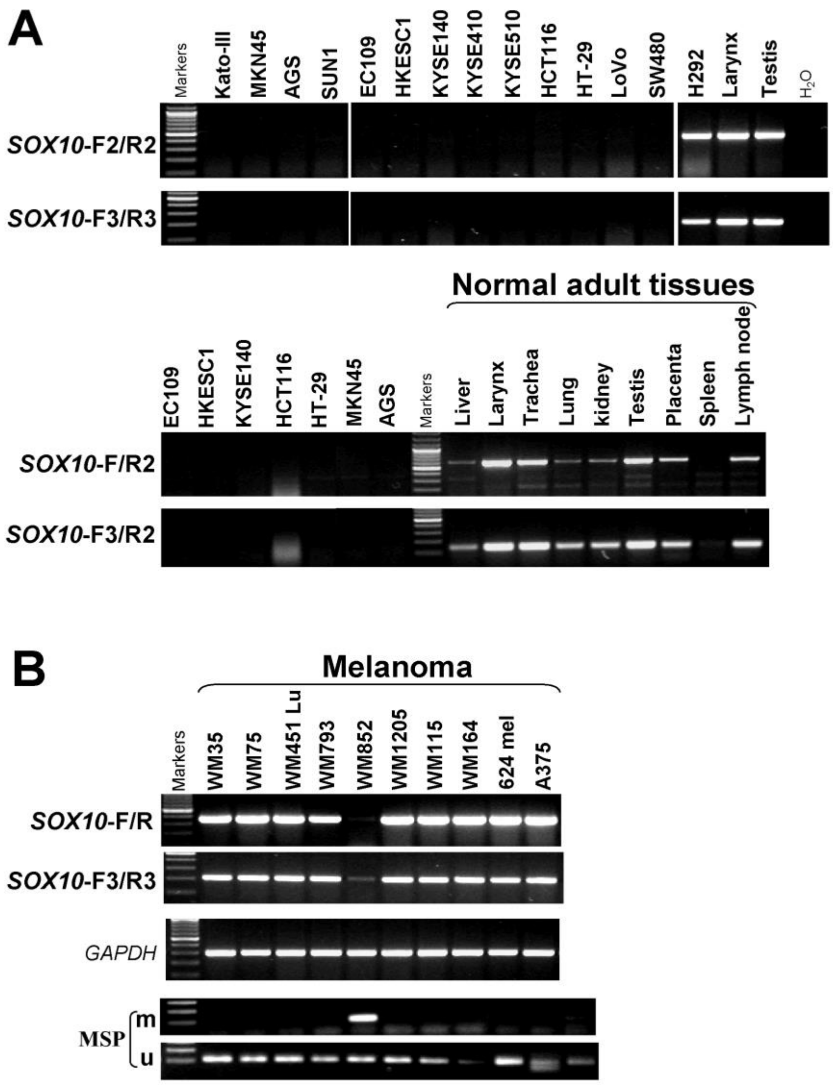

Supplemental Figure S1: Representative analyses of SOX10 expression and methylation in Lung (A) and melanoma carcinoma cell lines (B). Ca, carcinoma; M, methylated; U, unmethylated.

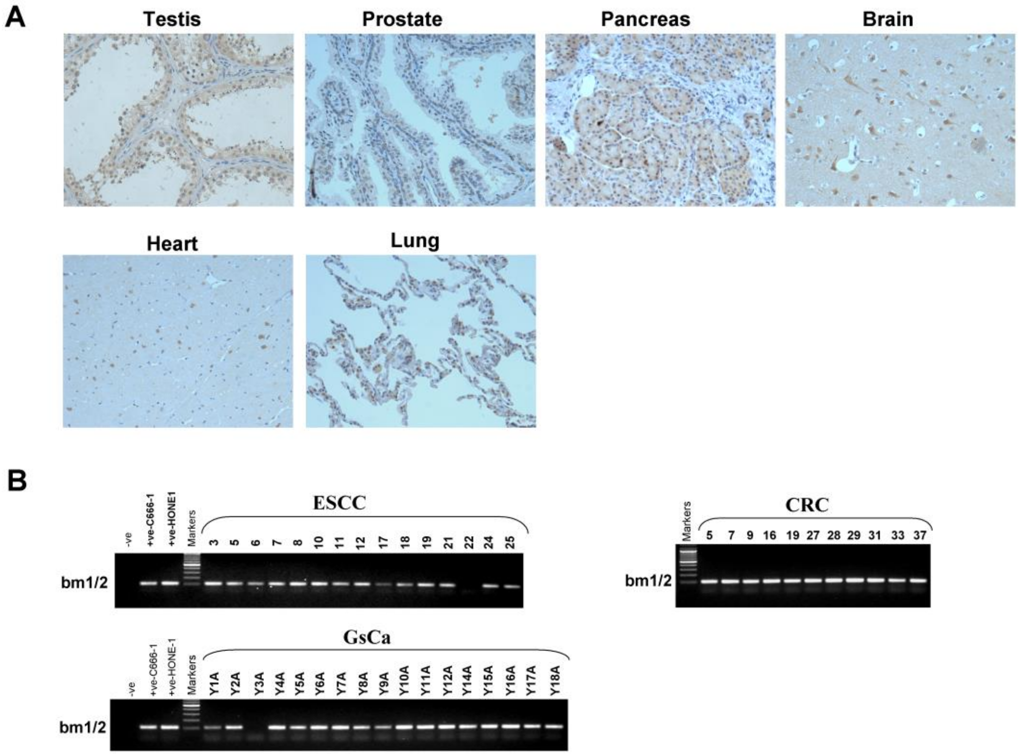

Supplemental Figure S2: (A) Representative immunostaining analyses of SOX10 expression in normal tissue. Original magnification, 200. (B) Representative analyses of SOX10 methylation in ESCC, Gsca and CRC tissue.

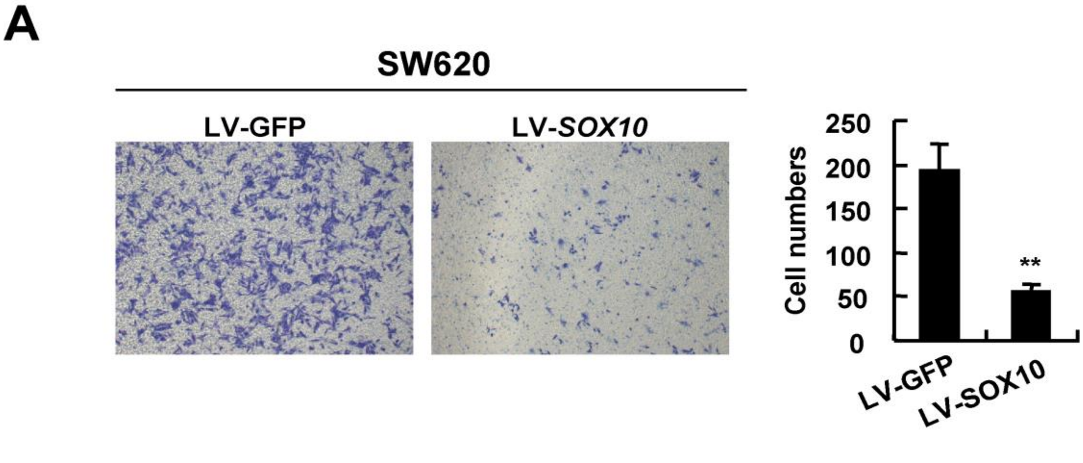

Supplemental Figure S3: Migration assay testing the ability of SW620 cells expressing GFP and SOX10 to migrate through transwell chambers.

References

1. Tao Q, Huang H, Geiman TM, Lim CY, Fu L, Qiu GH, Robertson KD. Defective de novo methylation of viral and cellular DNA sequences in ICF syndrome cells. Hum Mol Genet 2002;11:2091-102.

2. Tao Q, Swinnen LJ, Yang J, Srivastava G, Robertson KD, Ambinder RF. Methylation status of the Epstein-Barr virus major latent promoter C in iatrogenic B cell lymphoproliferative disease-Application of PCR-based analysis. American Journal of Pathology 1999;155:619-625.

3. Tong X, Li K, Luo Z, Lu B, Liu X, Wang T, Pang M, Liang B, Tan M, Wu M, Zhao J, Guo Y. Decreased TIP30 expression promotes tumor metastasis in lung cancer. Am J Pathol 2009;174:1931-9.
